# Nondestructive Classification Analysis of Green Coffee Beans by Using Near-Infrared Spectroscopy

**DOI:** 10.3390/foods8020082

**Published:** 2019-02-22

**Authors:** Naoya Okubo, Yohei Kurata

**Affiliations:** 1 Graduate School of Bioresource Sciences, Nihon University, 1866 Kameino, Fujisawa, Kanagawa 252-0880, Japan; currahee-506@hotmail.co.jp; 2 College of Bioresource Sciences, Nihon University, 1866 Kameino, Fujisawa, Kanagawa 252-0880, Japan

**Keywords:** near-infrared spectroscopy, green coffee beans, SIMCA

## Abstract

Near-infrared spectroscopy (NIRS) is a powerful tool for the nondestructive evaluation of organic materials, and it has found widespread use in a variety of industries. In the food industry, it is important to know the district in which a particular food was produced. Therefore, in this study, we focused on determining the production area (five areas and three districts) of green coffee beans using classification analysis and NIRS. Soft independent modeling of class analogy (SIMCA) was applied as the classification method. Samples of green coffee beans produced in seven locations—Cuba, Ethiopia, Indonesia (Bari, Java, and Sumatra), Tanzania, and Yemen—were analyzed. These regions were selected since green coffee beans from these locations are commonly sold in Japan supermarkets. A good classification result was obtained with SIMCA for the seven green bean samples, although some samples were partly classified into several categories. Then, the model distance values of SIMCA were calculated and compared. A few model distance values were ~10; such small values may be the reason for misclassification. However, over a 73% correct classification rate could be achieved for the different kinds of green coffee beans using NIRS.

## 1. Introduction

Near-infrared spectroscopy (NIRS) is an increasingly popular technique used for nondestructive evaluation. NIRS has found widespread use in a variety of industries, including the food, agricultural, pharmaceutical, and wood industries [1,2,3]. As a measurement technique, the advantages of NIRS include the rapid, easy, and nondestructive measurement process without the need for multiple chemical reagents. For recent NIRS research, the measurement methods and ways were developed like online measurement [4], portable measurement [5], and imaging analysis [6]. Its application range in food analysis continues to expand at an increasing rate. It probably becomes important as a process of quality control for food.

Coffee is one of the most popular beverages and an important farm product. Techniques to qualify and grade coffee beans have been developed and implemented. In particular, NIRS has been applied to assess several characteristics of coffee. Barbin et al. [7] extensively reviewed NIRS and its application to the evaluation of coffee bean properties. The discussion included the assignment of the near-infrared (NIR) spectra of coffee compositions, the assessment of coffee bean conditions, and NIR application techniques and analysis methods, such as multivariable analysis.

In this report, we focus on coffee conditions such as the coffee bean roast. Downey and Boussion [8] researched the authentication of the dried extract and lyophilized variety of roasted coffee beans using NIRS; beverages produced from pure arabica, pure robusta, and arabica robusta blends were discriminated with a high degree of success. Esteban-Díez et al. [9] measured the sensory properties of espresso from roasted coffee samples by NIRS. However, many of these studies examined roasted coffee, and few have evaluated green bean coffee samples.

Santos et al. [10] evaluated the quality of green coffee beans using NIRS by measuring the mass fractions of defective and nondefective beans. We also focused on green coffee beans, but we classified them according to their production areas by using NIRS. The ability to ascertain the production area prevents food fraud or contamination by coffee produced elsewhere. In particular, it is important in the Japanese food industry to know the production district of a particular food.

In this report, coffee beans from five areas and three districts were classified using NIRS. The NIR measurement mode was reflectance, and the wavelength range was 1200 to 2500 nm. Soft independent modeling of class analogy (SIMCA) was used for the classification analysis.

## 2. Material and Methods

### 2.1. NIR Measurement and Coffee Sample Preparation

The measurement device was NIR spectrometer S-7100 (Soma Optics Ltd., Tokyo, Japan). Reflectance mode was used for the light detection measurements. The NIR spectra were obtained at 1 nm resolutions over a wavelength range of 1200 to 2500 nm. To ensure signal stability, five scan data were averaged for each sample, using the same position for each measurement. NIR spectra were obtained from the center cutting face of the flat part of the green coffee bean, i.e., not the curved part.

Commercial green coffee beans were prepared from five areas and three districts that sell coffee to Japan: Cuba, Ethiopia, Indonesia (Bari, Java, and Sumatra), Tanzania, and Yemen. The samples were bought in supermarkets at Kanagawa prefecture in Japan. Faulty beans were considered to be contaminants, including beans damaged by insects or fungus and those with irregular shapes (e.g., peaberry), and were eliminated from the green coffee bean samples. Basic information on the samples used for the coffee analysis is summarized in Table 1. Although the samples originated from seven production areas, the breed variety was the Arabica species for all but one of the samples.

### 2.2. Data Analysis

The theory underlying SIMCA was discussed in a previous paper [11]. SIMCA is a classification analysis method that is often used with NIR spectroscopy [12,13,14,15]. SIMCA is a classification method based on principal component analysis (PCA). Spectra (in this research, test set data) of new samples to be classified are projected into the space of each model made by the training set. The distance to each individual model, expressed as the residual spectra variance, and the leverage of the new sample’s spectrum, compared to the mean leverage of each class, are used as the criteria to determine whether the new sample belongs to one class, to several classes, or to no class [16]. PCA involved several calculations utilizing orthogonalization procedures, such as singular-value decomposition, eigenvector calculation with the use of a covariant matrix, nonlinear iterative partial least squares, and successive average orthogonalization [17]. PCA models have a certain number of significant principal components (PCs). In this analysis, PCA was performed using a full cross-validation, and the outliers were eliminated. Outliers had lower absorbance than other samples because of less reflected light. The coffee bean shape of the center cutting face might be affected. The size of the training sets and the number of PCs for PCA of the measurements are summarized in Table 2. The final number of PCs was recommended by the Unscrambler software (Version 9.6; CAMO, Oslo, Norway).

Pretreatment NIR spectra data for the whole spectral range were used in PCA and SIMCA. The pretreatment were moving average, multiplicative scatter correction (MSC), and the second derivative. Moving average eliminates the random noise of NIR spectra. The MSC was used to compensate for both multiplicative and additive effects in the spectra. The second derivative also reduced the multiplicative and additive effects in the spectral data and increased peak amplification in the spectra. Pretreated NIR spectra (moving average smoothing with segment size 13, MSC, and the second derivative with the Norris Gap Derivative algorithm using a gap size of 13) were used for this analysis. Unscrambler software was used for spectral pretreatment and quantitative analysis with PCA and SIMCA. The classification results of SIMCA are shown at a significance level of 0.05 (α = 0.05).

## 3. Results and Discussion

**Figure 1:**
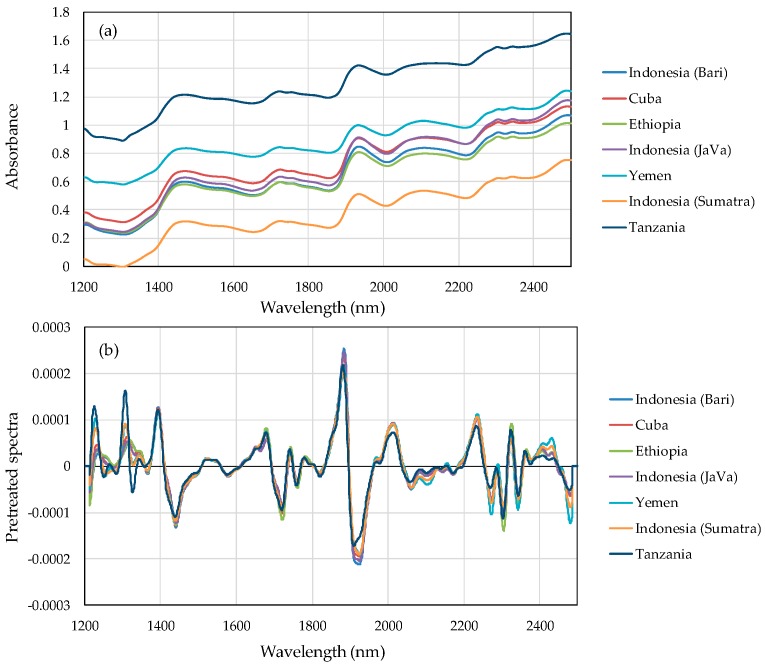
NIR spectra of each green coffee bean from the center cutting face: (**a**) raw spectra and (**b**) pretreated spectra by calculating averaged value per each wavelength.

Figure 1a plots NIR absorbance spectra and Figure 1b plots pretreatment spectra by calculating the averaged value per each wavelength in training set sample. The absorption was varied for the difference of each sample. In Figure 1b, the variation of several peaks are observed in the pretreated spectra in the 1200–1350 nm, 1850–1950 nm, and 2300–2500 nm regions.

**Figure 2:**
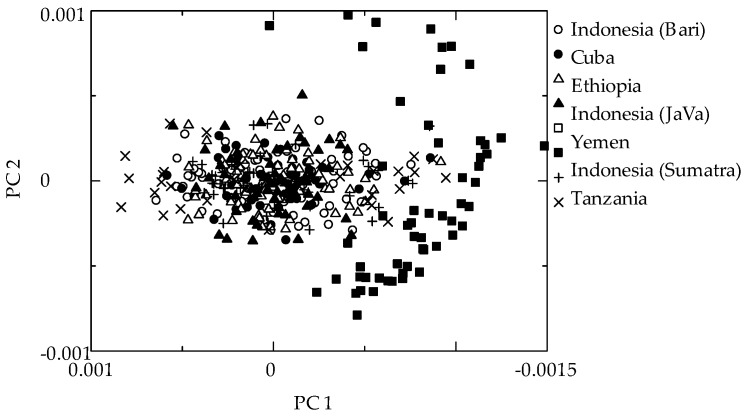
Score plot of PC1 versus PC2 for using pretreated NIR spectra.

Figure 2 shows the PC1 vs. PC2 score plot of the pretreated NIR spectra. Yemen samples had the different clustering, and clearly separated from the others. 

**Figure 3:**
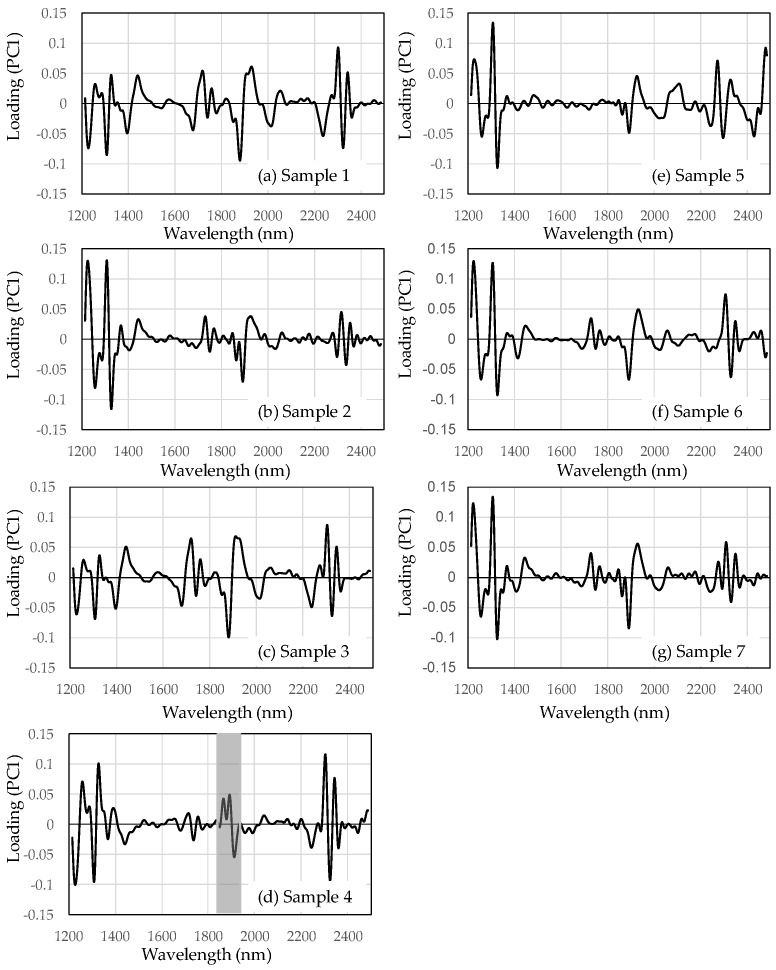
Loading plots of PC1 from seven different production areas: (**a**) Sample 1, (**b**) Sample 2, (**c**) Sample 3, (**d**) Sample 4, gray area indicates the characteristic peaks around 1850∓1950 nm,(**e**) Sample 5, (**f**) Sample 6, (**g**) Sample 7.

Figure 3 shows the loading plots of PC1 from seven different production areas. The loading plots of PC1 mainly indicate the absorption information of the green beans. For PC1 of sample 4, the trend at ~1850–1950 nm (Figure 3d, gray area) differs from the other samples for PC1. The NIR spectral region around 1850–1950 nm could be assigned to C=O, H_2_O, caffeine, chlorogenic acid, protein, lipids, and carbohydrates of the first overtone [7]. Many chemical compositions of coffee beans had absorption between 1850 and 1950 nm.

Table 3 shows the classification results of SIMCA for the green coffee beans at a significance level of 0.05 (α = 0.05). The best classification was obtained with sample 5 (Yemen), and the worst classification was found with sample 2. Sample 2 was classified into several categories with high percentages. There are a few misclassifications, i.e., assigning the wrong category, as well as instances with no category assigned. There was no difference detected among the coffee species in this sample combination. As 73% correct classification rate (sample 2) was not high classification result, the difference of production area with green coffee beans might be less. The chemical composition with production area might be changed by roasted procedure because of good results obtained roasted coffee beans [7,9].

**Figure 4:**
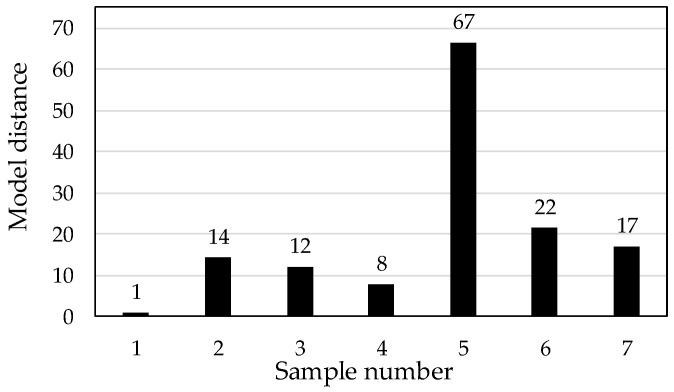
Model distance results of SIMCA for each sample (α = 0.05).

Additionally, we focused on the model distance from SIMCA for further discussion. This parameter indicates whether the models are really different. A value greater than 3 indicates that the models are significantly different [12]. Figure 4 shows the model distances from SIMCA for each green bean sample. The distance of the sample 1 model was assumed to have a value of 1. The model distance, excluding sample 5, is low, with a value of approximately 10–20. In particular, as sample 4 has a distance of 8, the different species—arabica and robusta—are similar in model distance. Such a low model distance value may be the reason for misclassifications. Sample 5 had the clearly separated from the others. Sample 5 might be different from other sample from PC1 vs. PC2 score plot, but to elucidate the difference was necessary to analyze the chemical compositions of the samples. Santos et al. [10] elucidated C–H and C–C combination bands absorption region (~2000–2500 nm) had the most information regarding the chemical composition of the defective and nondefective coffee beans. It might be compared directly because of the difference of measurement target. The chemical composition difference of coffee beans assigned NIR absorption between 1850 and 1950 and 2000 and 2500 were the presence of C=O and H_2_O absorption [7]. In this study, the presence of C=O and H_2_O absorption was useful to classify the production area for green coffee beans.

## 4. Conclusions

A nondestructive classification analysis for assessing the area of green coffee bean production was carried out by SIMCA. In this case, the NIR spectra were obtained from the flat surface of the seven different green beans. The classification analysis results of SIMCA are good; however, the model distance values from SIMCA are similar. For instance, as in samples 1 and 4, the different species—arabica and robusta—are similar in model distance (distance of 8). In results, the difference of production area or districts was more than that of coffee beans species. In this study, we did not perform chemical component analysis of the green beans. As the green coffee bean compositions might be similar, chemical compositions must be determined for further, deeper analysis and discussion. In this classification analysis, C=O and H_2_O absorption obtained from 1850 nm to 1950 nm might be important.

NIR spectra were measured from the center cutting face of the flat part of the green coffee bean. As the variance of absorbance was detected because of the different cutting face, such differed absorbance data were removed as outliers in this research. In practical use, as this measurement was highly time-consuming, the NIR image of the wavelength was limited in a way that was good for the measurements. However, NIRS represents a powerful nondestructive technique for the identification of green coffee beans.

## Figures and Tables

**Table 1:** Coffee sample information for analysis.

Sample Number	Country of Production (District)	Commodity Name	Breed Variety
1	Indonesia (Bari)	Bari	Arabica
2	Cuba	Crystal Mountain	Arabica
3	Ethiopia	Ethiopia	Arabica
4	Indonesia (JaVa)	Java	Robusta
5	Yemen	Mokha Mattari	Arabica
6	Indonesia (Sumatra)	Linton Mandheling	Arabica
7	Tanzania	Tanzania	Arabica

**Table 2:** Number or training set and principal components (PCs) for each measurement sample.

Sample Number	Country of Production (District)	No. in Training Set	No. of PCs
1	Indonesia (Bari)	73	6
2	Cuba	70	6
3	Ethiopia	76	6
4	Indonesia (JaVa)	57	7
5	Yemen	70	3
6	Indonesia (Sumatra)	48	6
7	Tanzania	51	4

**Table 3:** Soft independent modeling of class analogy (SIMCA) classification results (α = 0.05).

Sample	No. in Test Set	Correct Classification (%)	Classified Several Category (%)	Classified Wrong Category (%)	Classified No Category (%)
1	15	86	13	0	0
2	15	73	26	0	0
3	15	93	7	0	0
4	15	80	13	0	7
5	15	100	0	0	0
6	15	80	13	7	0
7	15	80	20	0	0
